# Approach-avoidance tendencies in depression and childhood trauma: No effect of noradrenergic stimulation

**DOI:** 10.1016/j.cpnec.2021.100077

**Published:** 2021-08-01

**Authors:** Christian Eric Deuter, Janna Smit, Michael Kaczmarczyk, Katja Wingenfeld, Christian Otte, Linn Kristina Kuehl

**Affiliations:** a Charité – Universitätsmedizin Berlin, Corporate Member of Freie Universität Berlin, Humboldt-Universität zu Berlin, And Berlin Institute of Health, Klinik für Psychiatrie und Psychotherapie, Campus Benjamin Franklin, Berlin, Germany; b Berlin Institute of Health (BIH), Berlin, Germany; c MSB Medical School Berlin, Germany

**Keywords:** Approach-avoidance, Emotional processing, Major depressive disorder, Adverse childhood experience, Yohimbine, Locus coeruleus, Noradrenaline, Norepinephrine

## Abstract

Adverse childhood experiences (ACE) are a major risk factor for major depressive disorder (MDD) in later life. Both conditions are characterized by dysregulations in the noradrenergic system related which again could represent a mediating mechanism for deficits in affective processing and behavioral functioning. In this double-blind, placebo-controlled study we tested the hypothesis that ACE and MDD are characterized by aberrant approach-avoidance (AA) tendencies and that these are mitigated after noradrenergic stimulation with yohimbine. In a mixed-measures, fully crossed design, participants (N = 131, 73 women) with/without MDD and with/without ACE received a single-dose of yohimbine or placebo on different days, followed by an AA task. We found modulation of AA tendencies by the emotional valence of target images, yet there were no effects of group or treatment. From these results, we conclude that AA tendencies are not critically affected by MDD or ACE and that the noradrenergic system is not substantially involved in this behavior.

## 1 Introduction

Motivated behavior is severely altered in patients with MDD, as expressed in social withdrawal, diminished approach to rewarding goals and lack of volition to evade unpleasant conditions. In typical behavioral paradigms for experimental assessment of approach-avoidance (AA) tendencies, participants are either required to pull/push a lever or joystick to increase/decrease the size of affective stimuli on a computer screen, usually words or images of facial expression, or to move a manikin or avatar on the screen towards (approach) or away (avoidance) from those target stimuli [10,12]. Healthy participants exhibit characteristic behavior, i.e. faster responses and lower error rates when approaching positive and avoiding negative stimuli. Previous studies found reduced approach behavior in MDD [1] or generally diminished AA tendencies [14], potentially reflecting a motivational process that could contribute to the pathogenesis of MDD. Adverse childhood experiences (ACE) are an important risk factor for MDD in later life [17] and contribute to aberrant AA tendencies as well [7]. ACE seems to generally increase avoidance tendencies, primarily to negative stimuli [8]. Patients suffering from MDD often report ACE, yet many studies conflate both.

Of note, both MDD and ACE are characterized by dysregulations of the locus coeruleus-noradrenergic system, which is also evident as a result of ACE [2,15]. While the pathophysiological mechanism behind these dysregulations is not fully understood yet, chronic stress during due to ACE could be a contributing developmental factor [15,17]. It has been shown that noradrenergic signaling and α-adrenergic receptor activity play a role for MDD patients’ cognitive and affective response patterns in different situations and paradigms [13,19]. However, to our knowledge, no study so far has investigated the role of noradrenaline for AA behavior in MDD and ACE. Therefore, we systematically varied these factors by including participants with/without MDD and with/without ACE and assessed the effect of a single dose of yohimbine – a α2 adrenoceptor antagonist that increases central availability of noradrenaline – on AA tendencies.

In the absence of our treatment and when compared to healthy controls, we predicted lower approach tendencies to positive stimuli in depressed patients. Further, we expected higher avoidance of negative stimuli in participants with ACE, when compared to those without ACE. These aberrant AA tendencies should both be present in the group that combined MDD and ACE. We hypothesized that these characteristic response patterns in MDD and ACE should be reduced following the administration of yohimbine, i.e. we expected significant between-group effects for the placebo condition which should have been reduced or absent after our treatment.

## 2 Methods

We subjected 138 participants to this double-blind, placebo-controlled design, seven participants did not provide full data sets or were excluded for technical reasons. All participants provided informed written consent, the study was approved of by the local ethical commission. Participants received a single dose of 10 mg yohimbine and placebo on two separate days in quasi-randomized order. Patients fulfilled the criteria for an acute episode of MDD; ACE was defined as repeated physical or sexual abuse at least once a month over one year or more until the age of 18. Both were assessed in structured clinical interviews. The AA task was part of an extended study setup; the results of other tasks and the general methods (participant and demographic characteristics, general procedure, assessment and analysis of blood pressure and salivary alpha-amylase) have been reported in detail elsewhere [4,11,16].

**Fig. 1:**
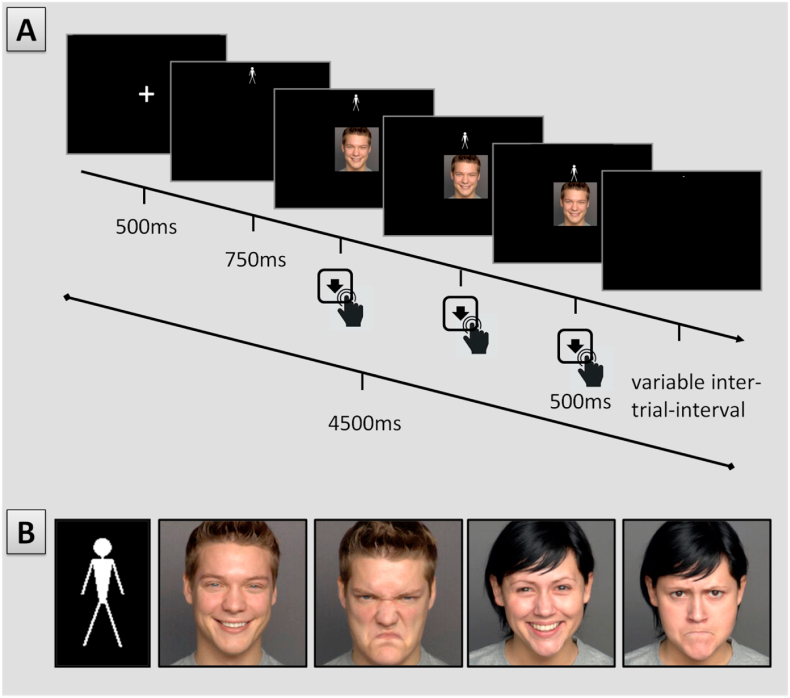
(A) Sample trial from the congruent condition. In this case, the manikin had to be moved towards a happy face by three consecutive button presses of the arrow key. After full approach, the slide remained for 500 ms. The manikin could appear above or below the face picture in random order. For avoidance, the manikin had to be moved away from the face to the upper or lower edge of the screen. Responses in the wrong direction and no responses for 1500 ms were considered errors. (B) The manikin (left) and sample pictures of happy and angry, male and female faces. Each model appeared in both expressions. The order of valence and sex was randomized.

We assessed AA tendencies using a task developed by Ref. [3] and later validated by Krieglmeyer et al.(2010). Image stimuli were obtained from the FACES database [5]. In this task, participants had to move a manikin either towards (approach) or away (avoid) from a target image depicting an angry or happy facial expression by pressing the up- or down arrow keys on a standard keyboard. They were instructed to imagine that they themselves were the manikin and to respond as fast and accurately as possible. Two blocked conditions were implemented: in the congruent response condition, participants had to approach happy and avoid angry faces; this pattern was reversed in the incongruent condition. The order of blocks was counterbalanced between participants. Emotional expression varied trial-wise (see Fig. 1).

Reaction times (time from image onset to final manikin position), response latency (image onset to first button press) and accuracy (percent of correct responses) were taken as outcome measures. Data were analyzed by conducting mixed-measures ANOVAS, with the within-subject factors ‘emotion’ (happy vs. angry), ‘AA’ (approach vs. avoidance) and ‘drug’ (placebo vs. yohimbine) and the between-subjects factors MDD and ACE, *p* < .05 was considered to indicate statistical significance. Data analyses were carried out using SPSS 22.0.

## 3 Results

We found an interaction between ‘Emotion x AA’ for all outcome measures: in the congruent condition (approaching positive and avoiding negative stimuli), participants made more correct and faster responses, i.e. these were initiated at shorter latency and reached the final position faster, compared to the incongruent condition. However, we found no main effects or interactions for any outcome that included the experimental manipulation ‘drug’, or the group factors ‘MDD’ or ‘ACE’: yohimbine did not affect reaction times, response latency or accuracy and we also did not find significant group effects in these measures (all *p* > .05, see Table 1).

**Table 1:** Mean scores and standard deviations (SD) for each group and results of statistical analysis. Abbr.: Emo = Emotion (happy vs. angry), AA = Approach vs. Avoidance, MDD = Major depressive disorder, ACE = Adverse childhood experience, * - indicates statistical significance.

					**MDD+/ACE+** (n = 27)	**MDD+/ACE-**(n = 26)	**MDD-/ACE+** (n = 30)	**MDD-/ACE-**(n = 48)	***F***	***df***	***p***	**eta**_**p**_^**2**^
**Reaction Time**	Statistics	Emo x AA*							85.33	1,127	<.001	0.400
		Emo x AA x MDD							0.08	1,127	.78	0.001
		Emo x AA x ACE							0.14	1,127	.71	0.001
		Emo x AA x MDD x ACE							1.20	1,127	.28	0.009
		Emo x AA x Drug							0.26	1,127	.61	0.002
		Emo x AA x Drug x MDD							0.85	1,127	.36	0.007
		Emo x AA x Drug x ACE							<0.01	1.127	,97	<0.001
		Emo x AA x Drug x MDD x ACE							0.45	1.127	,50	0.004
	Time in ms, Mean (SD)	Placebo	approach happy		2970.42 (541.57)	2840.07 (600.40)	2769.89 (555.26)	2702.79 (560.50)				
			avoid happy		3246.78 (584.05)	3099.48 (648.81)	3109.12 (600.24)	2940.57 (552.88)				
			approach angry		3111.57 (602.09)	3014.73 (662.95)	3015.09 (639.51)	2836.39 (575.87)				
			avoid angry		3138.63 (484.19)	2901.73 (549.43)	2946.29 (530.81)	2802.87 (497.16)				
		Yohimbine	approach happy		2970.42 (541.57)	2840.07 (600.40)	2769.89 (555.26)	2702.79 (560.50)				
			avoid happy		3246.78 (584.05)	3099.48 (648.81)	3109.12 (600.24)	2940.57 (552.88)				
			approach angry		3111.57 (602.09)	3014.73 (662.95)	3015.09 (639.51)	2836.39 (575.87)				
			avoid angry		3138.63 (484.19)	2901.73 (549.43)	2946.29 (530.81)	2802.87 (497.16)				
**Response delay**	Statistics	Emo x AA*							83.65	1,127	<.001	0.397
		Emo x AA x MDD							0.09	1,127	.76	0.001
		Emo x AA x ACE							0.25	1,127	.62	0.002
		Emo x AA x MDD x ACE							0.61	1,127	.44	0.005
		Emo x AA x Drug							0.20	1,127	.66	0.005
		Emo x AA x Drug x MDD							0.30	1.127	,59	0.002
		Emo x AA x Drug x ACE							<0.01	1,127	.97	<0.001
		Emo x AA x Drug x MDD x ACE							0.57	1.127	,45	0.004
	Time in ms, Mean (SD)	Placebo	approach happy		784.61 (161.53)	748.15 (182.10)	728.46 (168.45)	709.85 (176.30)				
			avoid happy		877.52 (180.04)	831.50 (190.98)	836.38 (184.82)	786.21 (170.43)				
			approach angry		836.85 (191.96)	806.48 (197.82)	804.19 (201.60)	752.65 (179.36)				
			avoid angry		840.08 (144.97)	768.46 (156.32)	783.76 (156.18)	741.78 (151.49)				
		Yohimbine	approach happy		762.78 (168.31)	733.56 (153.78)	736.75 (178.30)	706.27 (173.13)				
			avoid happy		863.47 (162.84)	809.05 (172.44)	818.97 (159.13)	784.59 (171.52)				
			approach angry		829.06 (171.56)	802.19 (193.12)	792.61 (167.55)	758.49 (176.51)				
			avoid angry		824.39 (145.05)	769.09 (150.33)	776.15 (162.08)	754.26 (153.61)				
**Percent correct respones**	Statistics	Emo x AA*							24.93	1,127	<.001	0.160
		Emo x AA x MDD							3.35	1,127	.07	0.026
		Emo x AA x ACE							2.69	1,127	.10	0.021
		Emo x AA x MDD x ACE							1.16	1,127	.28	0.009
		Emo x AA x Drug							0.88	1,127	.35	0.007
		Emo x AA x Drug x MDD							1.49	1.127	,22	0.012
		Emo x AA x Drug x ACE							0.19	1,127	.66	0.002
		Emo x AA x Drug x MDD x ACE							3.12	1.127	,08	0.024
	Percent, Mean (SD)	Placebo	approach happy		96.22 (5.51)	96.55 (4.25)	95.83 (6.66)	96.96 (5.72)				
			avoid happy		95.52 (4.72)	92.07 (12.35)	94.58 (8.76)	96.74 (3.74)				
			approach angry		93.90 (7.84)	90.38 (13.30)	95.90 (6.46)	96.27 (5.14)				
			avoid angry		95.29 (5.85)	94.55 (6.15)	96.11 **(**5.52)	96.92 (4.05)				
		Yohimbine	approach happy		96.22 (4.44)	96.39 (5.39)	96.74 (5.09)	97.22 (3.85)				
			avoid happy		94.37 (7.46)	94.31 (6.73)	95.76 (5.06)	95.66 (6.46)				
			approach angry		93.67 (11.75)	92.63 (9.49)	94.03 (8.36)	94.44 (7.95)				
			avoid angry		95.60 (5.34)	96.71 (3.92)	95.42 (4.75)	97.53 (3.43)				

## 4 Discussion

We could replicate the basic AA effect, with facilitated approach to positive and avoidance of negative stimuli, and could therefore confirm the validity of the paradigm. In contrast to our hypotheses, the results could not demonstrate an effect of yohimbine on AA tendencies in MDD and ACE. In fact, these groups did not deviate from healthy controls, irrespective of our intervention.

Possible explanations for the divergent findings in previous studies could be both in different paradigms and differences in recruitment. Analogous to Ref. [14] we used happy and angry facial expressions as negative stimuli. These stimuli have high ecological validity in the presence of high social anxiety, but may not be optimally suited to the specific mood state of patients with MDD. Future studies could use emotional pictures from the International Affective Picture System [1] or sad facial expressions in a similar setting, with “sadness” having a closer conceptual proximity to depression [9]. However, potentially expressions of sadness evoke different approach vs. avoidance patterns from expressions of anger or other negative stimuli, such as the tendency to help the sad person and provide comfort, which certainly would need to be taken into account. The effects of ACE also might have been more prominent with trauma-specific stimuli. Also, some previous studies used a different task response layout, which requires the participant to pull or push a joystick lever to increase or decrease the size of the image. While the task that we employed is better suited to assess AA tendencies in a general population [10], this might not be the case for the clinical groups that we investigated. Further, other studies found effects of MDD when taking other factors such as rumination [6] into account, which suggests that these effects are restricted to specific subgroups. In contrast to many previous studies, patients in our study were tested directly before the start of pharmacological treatment and received no antidepressant medication. Our sample size was large enough to detect effects with a medium to small effect size (f = 0.175). Although previous studies describe effects of MDD in this order of magnitude we would like to point out that findings in this field are heterogeneous, with a recent and sufficiently sample-sized study also reporting no effect of MDD on AA behavior [18]. We conclude from our results that aberrant AA tendencies might not be a characteristic feature in MDD or ACE and therefore might not serve as an explanatory mechanism for affective and behavioral dysfunctions in these populations. As a limitation, we would like to point out that this is a cross-sectional study in adults, with the consequence that both ACE, and in many cases the onset of MDD, occurred decades ago. Whether this has an influence on the examined measures we cannot answer with this design. A reasonable extension would be the investigation in an adolescent sample or a longitudinal study with the aim to investigate the consequences of ACE later in life.

## Declaration of competing interest

The authors declare that they have no known competing financial interests or personal relationships that could have appeared to influence the work reported in this paper.
